# Evaluation of the efficacy of different lung biopsy modalities in solitary pulmonary nodules

**DOI:** 10.3389/fbioe.2026.1743070

**Published:** 2026-04-22

**Authors:** Wei Zhao, Chen Liu, Yajie Hu, Yanhong Du, Yunhui Zhang

**Affiliations:** 1 Department of Respiratory Medicine, The First People’s Hospital of Yunnan Province, Kunming, China; 2 The Affiliated Hospital of Kunming University of Science and Technology, Kunming, Yunnan, China

**Keywords:** biopsy, needle, bronchoscopy, navigation systems, solitary pulmonary nodule, tomography, X-ray computed

## Abstract

**Introduction:**

This study compared the diagnostic performance and safety of computed tomography (CT)-guided percutaneous transthoracic needle biopsy (PTNB) and virtual bronchoscopic navigation (VBN)-guided transbronchial lung biopsy (TBLB) in patients with solitary pulmonary nodules (SPNs).

**Methods:**

A retrospective analysis was conducted on 204 SPN patients, divided into CT-guided (n = 102) and VBN-guided (n = 102) groups. Parameters, including diagnostic yield, procedure time, and complication rates, were compared between the two methods.

**Results:**

After applying rigorous diagnostic criteria per ATS/ACCP guidelines, the overall diagnostic yield was higher in the VBN group (64.7%, 66/102) than in the CT group (59.8%, 61/102), though the difference was not statistically significant (P = 0.472). In exploratory subgroup analyses, VBN demonstrated higher diagnostic yield for smaller nodules (8–20 mm) and those located closer to the pleura (2–3 cm) (both P < 0.05). For larger nodules (20–30 mm) or those further from the pleura (3–5 cm), diagnostic rates were comparable between groups. The VBN group had significantly shorter procedure times, including lesion localization (6.62 ± 0.93 vs. 9.27 ± 1.05 min) and total procedure duration (22.04 ± 2.36 vs. 38.16 ± 4.29 min) (P < 0.01). The complication rate was significantly lower in the VBN group (9.80% vs. 29.41%, P < 0.01), with the CT group showing higher incidences of pneumothorax, hemoptysis, and chest pain in nodules 2-3 cm from the pleura. Multivariate analysis identified several factors significantly associated with diagnostic outcomes. Additionally, the VBN group incurred lower medical costs and shorter hospital stays (P < 0.01).

**Conclusion:**

VBN-guided biopsy was associated with higher diagnostic yield, fewer complications, shorter procedure times, and lower costs compared to CT-guided biopsy. These findings warrant confirmation in prospective randomized trials.

## 1 Introduction

Lung cancer remains one of the most prevalent and lethal malignancies globally (Wahidi et al., 2007), with increasing incidence and mortality rates, particularly in developing countries such as China (Wu et al., 2021; Liu et al., 2023). It primarily affects older adults, with the majority of cases diagnosed in individuals over 60 years of age. Common clinical symptoms include cough, dyspnea, hemoptysis, and chest pain, though early-stage lung cancer may be asymptomatic (Bade and Dela Cruz, 2020). Screening programs using low-dose computed tomography (CT) have been shown to reduce lung cancer mortality by enabling early detection of malignant solitary pulmonary nodules (SPNs) (Oliver, 2022; Liu et al., 2022). Clinical studies indicate that 80%–90% of small pulmonary nodules detected by chest CT are benign, although some may progress to malignancy over time (Zhu et al., 2023). Research by Kim et al. suggests that early-stage lung cancer offers a favorable prognosis, with a 5-year survival rate of 92%. Nodules smaller than 1 cm have a malignancy probability of 6%–28% (Yan et al., 2017), while those larger than 2 cm have a malignancy rate of 64%–82% (Gibson et al., 2017). Differentiation from other pathologies depends on factors such as SPN morphology, size, calcification pattern, growth, and margin characteristics. An SPN is defined as a solitary, well-defined, round, opaque nodule ≤3 cm in diameter, surrounded by lung parenchyma, not adjacent to the hilum or thoracic septum, and without associated atelectasis or pleural effusion. Lesions greater than 3 cm are classified as masses, with a higher malignancy likelihood, though benign masses are also possible (Chen et al., 2022).

Early lung cancer screening techniques, such as low-dose CT, have proven effective in reducing mortality by enhancing early detection of malignant SPNs (Oliver, 2022). In clinical practice, SPNs often require further diagnostic evaluation to confirm malignancy (Mattox et al., 2023). CT-guided percutaneous transthoracic needle biopsy (CT-PTNB) is a minimally invasive procedure offering an alternative to transthoracic surgical excisional biopsy. Its diagnostic efficacy for SPNs has been extensively validated in both national and international studies (Kim et al., 2020; Atkins et al., 2020). Bronchoscopy is suitable primarily for lesions with a leading bronchus, while many peripheral nodules, especially those in the upper lobes, are more accessible through CT-PTNB. Conventional diagnostic methods often have limitations due to lesion size and location. However, advancements in imaging-guided and navigational techniques, such as X-ray guidance (Borgheresi et al., 2023), ultrathin bronchoscopy (Gibson et al., 2017), fluorescence confocal microscopy (FCFM) (Atkins et al., 2020), endobronchial ultrasound with guide sheath (EBUS-GS) (Xu et al., 2021; Zhang et al., 2024), electromagnetic navigation bronchoscopy (ENB) (Alshari et al., 2020), virtual bronchoscopic navigation (VBN) (Choi et al., 2016), and endobronchial ultrasound with guide sheath (EBS) (Xu et al., 2021; Miotto et al., 2023), have improved diagnostic outcomes for SPNs. As VBN and similar bronchoscopic techniques gain wider adoption, the diagnostic success of SPN biopsies has significantly improved, although further research is still needed (Wu et al., 2021). The present study aims to evaluate the diagnostic value of CT-guided PTNB and VBN-guided transbronchial lung biopsy (TBLB) in differentiating benign from malignant SPNs, offering insights for optimizing biopsy method selection, enhancing positivity rates, and minimizing procedural risks.

## 2 Materials and methods

### 2.1 General information

**FIGURE 1:**
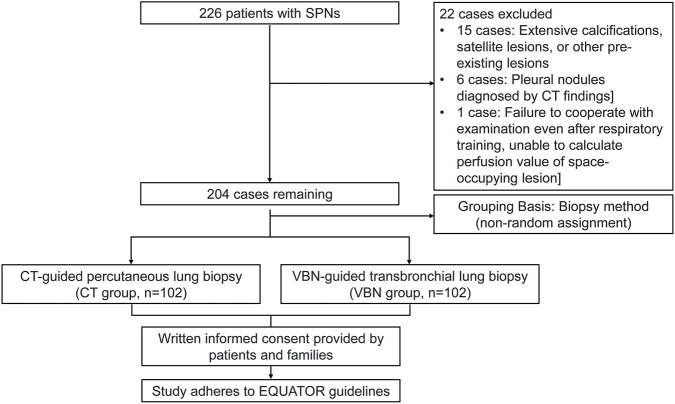
Flowchart of the study.

A retrospective analysis was conducted on 226 patients with SPNs who received treatment at the First People’s Hospital of Yunnan Province from January 2018 to January 2022. Twenty-two patients were excluded from the study: 15 had extensive calcifications, satellite lesions, or other pre-existing lesions; six were diagnosed with pleural nodules based on CT findings; and one patient was excluded due to inability to cooperate with the procedure following respiratory training. The remaining 204 patients were divided into two groups based on biopsy method: the CT-guided percutaneous lung biopsy group (CT group, n = 102) and the VBN-guided TBLB group (VBN group, n = 102). This study adhered to the EQUATOR guidelines. Prior to biopsy, all patients were fully informed of the associated risks and precautions, and written informed consent was obtained from both patients and their families. This non-randomized, real-world allocation reflects routine clinical practice, where the choice of biopsy method is influenced by nodule characteristics and patient-specific factors. Baseline differences between the groups were anticipated and were addressed using multivariate adjustment in the analysis. This design resulted in baseline differences between groups, which were adjusted for in subsequent multivariable analyses (Figure 1).

### 2.2 Inclusion criteria

Inclusion criteria were as follows: (Wahidi et al., 2007): Pulmonary nodules with a diameter between 8 and 30 mm, detected by chest X-ray or CT; (Wu et al., 2021); Nodules that might contain small cavities or calcifications; (Liu et al., 2023); Absence of mediastinal or hilar lymph node enlargement.

### 2.3 Exclusion criteria

Exclusion criteria were as follows: (Wahidi et al., 2007): Presence of extensive calcifications, satellite lesions, or other pre-existing lesions; (Wu et al., 2021); Nodules identified as pleural in nature, as indicated by CT findings showing proximity to and broad attachment with the pleura; (Liu et al., 2023); Patients unable to cooperate with the procedure after respiratory training.

### 2.4 Data collection

In addition to conventional baseline data, biopsy site, nodule size, diagnostic rate, pathological results, operation time, distance from the lesion to the pleura, and complication, the following additional information was collected:

Clinical treatment modality: Data on treatment plans post-diagnosis, including surgery, radiotherapy, chemotherapy, observation, and follow-up.

Diagnosis time: The duration from the start of examination to definitive diagnosis.

Patient medical costs: Itemized expenses, including examination, treatment, and hospitalization fees.

Operational difficulty assessment: A quantitative score based on physicians’ operational records and feedback to evaluate the difficulty of each biopsy method.

Operator experience was categorized as ‘Junior’ (less than 5 years of independent practice in interventional pulmonology) or ‘Senior’ (≥5 years).

### 2.5 Research methodology

CT group: The CT scan confirmed precise needle tip positioning within the lesion. The biopsy gun was activated to obtain a single tissue sample, after which the needle was retracted, and the sample were carefully extracted. The tissue was placed in a fixative within the biopsy specimen container and sent for pathological analysis. A smear was also prepared for microbiological and cytological examination. A follow-up CT scan was performed to assess for complications such as pneumothorax or hemorrhage. The biopsy site was disinfected, covered with a sterile dressing, and secured with adhesive tape. All CT examinations were performed using a 64-slice multidetector CT scanner (Philips Brilliance 64, Philips Healthcare, United States). The scanning parameters were as follows: tube voltage 120 kVp, tube current 50–100 mAs (with automated tube current modulation based on patient habitus), beam collimation 64 mm × 0.625 mm, pitch 0.5–0.8, rotation time 0.5 s, and slice thickness 1.0 mm with a reconstruction interval of 0.7–1.0 mm. All images were reconstructed using both standard soft tissue and lung kernels, and iterative reconstruction was applied to reduce radiation dose while maintaining image quality.

VBN group: DICOM data from post-HRCT scans were imported into a computer using VBN software, which generated a virtual tracheobronchial model of the target lesion. The VBN system identified the optimal path to the lesion, marking bronchial bifurcations to guide the bronchoscope toward peripheral lung lesions. Upon confirming proximity to the target using hypoechoic ultrasound, the ultrasound probe was slowly withdrawn to measure the bronchial distance to the lesion. Based on this measurement, biopsy forceps were introduced into the bronchus after the probe was retracted, and tissue samples were collected at the ultrasonically localized lesion site, with five specimens taken from each targeted area. The VBN system used was the LungPoint virtual bronchoscopic navigation system (Broncus Medical, Inc., United States), version 4. This system imports chest CT data in DICOM format to reconstruct a virtual tracheobronchial tree, automatically identifies the optimal pathway to the target lesion, and provides real-time navigation guidance during the bronchoscopy procedure.

Percutaneous lung biopsy by aspiration: Based on the pre-puncture CT imaging, the puncture route was planned, and the patient was positioned (lying, lateral, or prone) to balance comfort and optimal access for the procedure. The skin was marked, stabilized with a custom-made fence locator, and a CT scan was performed to select the ideal biopsy level. The route was designed to avoid major blood vessels, alveoli, and necrotic areas. The puncture site was marked, disinfected with iodophor, and anesthetized with 2% lidocaine, including local infiltration of the pleura. A 17G coaxial puncture needle was inserted into the extrapleural area following the pre-marked angle and depth. A second CT scan confirmed needle alignment; once correct positioning was verified, the needle was advanced to the lesion’s edge. For lesions further from the pleura or smaller in size, a gradual approach was used, advancing the coaxial needle incrementally. A subsequent CT scan confirmed ideal positioning of the cutting groove, after which the semi-automatic biopsy needle was activated to obtain the sample. The tissue was placed in formalin. Following needle retraction, the coaxial core was replaced, and a final CT scan was performed to detect any complications.

Total procedure time was defined as the interval from the initiation of anesthesia (local anesthesia for CT-guided biopsy; conscious sedation for VBN-guided bronchoscopy) to the completion of specimen collection. For the CT-guided biopsy, this included patient positioning, CT scanning, needle placement, and tissue sampling. For the VBN-guided biopsy, it encompassed navigation, ultrasound localization, and forceps biopsy. Lesion localization time was recorded separately as the time from the start of navigation (VBN) or the first CT scan (CT) to the confirmation of the needle/forceps position at the lesion.

Specimen handling: The entire procedure followed the histopathological and microbiological specimen handling protocols established by our research center. Cytological smears were prepared by three qualified, experienced pathologists. Specimens were delivered within 30 min and processed within 2 h. Tissue samples were fixed, paraffin-embedded, and sectioned for pathological analysis. In cases where the biopsy suggested metastatic lung malignancy, a comprehensive diagnosis was made by comparing the biopsy with pathology from the primary site. Special immunohistochemical tests were conducted as needed to refine the diagnosis. Depending on the patient’s condition, the operating physician determined if additional microbiological tests (such as Gram stain, acid-fast stain, *Mycobacterium tuberculosis* PCR, and bacterial/fungal cultures with drug sensitivity) should be performed on the bronchoscopic biopsy specimen for diagnostic support.

Specimen interpretation: In this study, a specimen was considered pathologically positive for malignant neoplasm upon the microscopic identification of cancerous cells. Nodular disease was confirmed when epithelioid cells or granulomatous nodules were observed. Tuberculosis was diagnosed based on the presence of exudative lesions or caseous necrosis, positive acid-fast staining on tissue smears, or positive bacterial culture with colony growth. Negative results were interpreted as chronic inflammation or other non-malignant conditions, confirmed through follow-up or surgical procedures. A definitive diagnosis was defined as a specific benign or malignant entity confirmed by histopathology, microbiology, or surgical resection. Non-specific findings such as ‘chronic inflammation’ or ‘lung inflammation’ were considered non-diagnostic. According to ATS/ACCP guidelines, radiological follow-up alone does not constitute a definitive benign diagnosis, and such cases were classified as non-diagnostic. Cases lost to follow-up without a definitive diagnosis were also classified as non-diagnostic.

### 2.6 Observation indicators

In this study, the observation indicators were expanded beyond the conventional ones to align with the updated research objectives. These indicators now include:

1. Diagnostic-related indicators: Diagnostic rate, biopsy site, and nodule size, which remain essential for evaluating the fundamental diagnostic performance of the two biopsy methods.
2. Time-related indicators: Total biopsy procedure time, lesion localization time, and diagnostic time (the duration from the initiation of examination to the final diagnosis), aiming to assess the efficiency of the two methods in the diagnostic process.
3. Complication-related indicators: The presence of complications during and after the biopsy procedures, with detailed records of specific symptoms such as anesthesia allergy, hemoptysis, hypoxemia, pneumothorax, and chest pain.
4. Clinical treatment-related indicators: The distribution of treatment modalities adopted post-diagnosis, including surgery, radiotherapy, chemotherapy, observation, and follow-up, to analyze how different biopsy methods influence subsequent treatment decisions.
5. Economic indicators: Patient medical cost details, covering examination, treatment, hospitalization, and other related expenses, for conducting a cost-effectiveness analysis.
6. Operational difficulty indicators: Quantitative scores assessing operational difficulty based on physicians’ records and feedback, facilitating a comparison of the practical challenges associated with each biopsy method.

### 2.7 Associated complications and management

During the biopsy procedures, patients were closely monitored for signs of anesthesia allergy, hemoptysis, hypoxemia, pneumothorax, chest pain, or other abnormal symptoms. Post-procedure, patients were instructed to fast for 2 h and were subjected to electrocardiographic monitoring to continuously track heart rate, blood pressure, and peripheral oxygen saturation. Symptoms such as hemoptysis, chest pain, chest tightness, or dyspnea were continuously observed.

Emergency protocols were established as follows:

1. For allergic reactions to anesthetics, standard drug allergy management protocols were strictly followed.
2. In the case of minor hemoptysis, continuous suction was performed until the symptom resolved spontaneously. For moderate hemoptysis, the bronchoscope lens was blocked in the biopsied lobe, and local ice saline or epinephrine spray was administered. In cases of massive hemoptysis, measures such as balloon occlusion, catheter placement, possible surgical intervention, systemic coagulants, and intubation, when necessary, were employed.
3. When hypoxia and cyanosis occurred during bronchoscopy, the procedure was immediately terminated.
4. The management of pneumothorax varied according to its severity. For mild pneumothorax, bed rest and high-concentration oxygen inhalation were provided; for moderate pneumothorax, high-concentration oxygen inhalation, chest biopsy puncture with aspiration or closed-chest drainage, and sedation/analgesia as required were carried out. Severe pneumothorax generally required high-concentration oxygen inhalation, closed-chest drainage, and sedation with analgesia.

### 2.8 Statistical analysis

Statistical analyses were conducted using SPSS 22.0. Continuous variables with a normal distribution were presented as mean ± standard deviation and compared using independent-sample t-tests. Categorical variables were expressed as counts and percentages, and analyzed using chi-square tests or Fisher’s exact test, as appropriate. Multivariate logistic regression was employed to identify factors independently associated with diagnostic success, with results presented as odds ratios (OR) and 95% confidence intervals (CI). A two-sided P-value <0.05 was considered statistically significant. To control for potential confounders due to the non-randomized design, multivariate logistic regression was performed, incorporating all variables showing significant between-group differences in baseline characteristics (age, nodule size, distance to pleura, nodule density, smoking history, lesion location, and operator experience) as covariates. Variables with P < 0.10 in univariate analysis were entered into the multivariate logistic regression model using a forward stepwise selection procedure. Age was modeled as a categorical variable (≥65 vs. <65 years) based on clinical relevance.

## 3 Results

### 3.1 General clinical information

The baseline characteristics of the two groups were compared (Table 1). The CT group consisted of 54 males and 48 females, with a mean age of 65.21 ± 9.54 years, while the VBN group included 47 males and 55 females, with a mean age of 54.15 ± 11.90 years. Among the 204 included patients, 5 (2.5%) were lost to follow-up (3 in CT group, 2 in VBN group) and were classified as non-diagnostic. The remaining 199 patients had complete follow-up. After applying ATS/ACCP criteria, 127 (63.8%) obtained a definitive diagnosis through biopsy (61 in CT group, 66 in VBN group), while 72 (36.2%) had non-diagnostic results (including 43 cases with non-specific inflammation and 29 cases with inconclusive findings despite follow-up).

**TABLE 1:** Baseline characteristics comparison.

Variable	CT group (n = 102)	VBN group (n = 102)	χ^2^/t	P-value
Sex, [n (%)]	​	​	0.962	0.326
Female	48 (47.1%)	55 (53.9%)	​	​
Male	54 (52.9%)	47 (46.1%)	​	​
Age (years, mean ± SD)	65.21 ± 9.54	54.15 ± 11.90	1.216	0.226
BMI (kg/m^2^, mean ± SD)	22.27 ± 2.83	22.39 ± 2.92	0.068	0.941
Nodule location, [n (%)]	​	​	4.152	0.386
Left upper lobe	23 (22.5%)	18 (17.6%)	​	​
Left lower lobe	17 (16.7%)	23 (22.5%)	​	​
Right upper lobe	33 (32.4%)	33 (32.4%)	​	​
Right middle lobe	12 (11.8%)	6 (5.9%)	​	​
Right lower lobe	17 (16.7%)	22 (21.6%)	​	​
Serum CEA (ng/mL, median [IQR])	2.51 ± 2.15	2.67 ± 2.78	−0.449	0.654
WBC (10^9^/L)	6.39 ± 1.89	6.54 ± 2.12	0.381	0.703
Neutrophil (10^9^/L)	4.05 ± 1.63	4.03 ± 1.82	0.059	0.952
RBC (10^12^/L)	4.65 ± 0.59	4.84 ± 0.67	1.537	0.127
HG (g/L)	138.98 ± 16.12	143.94 ± 16.48	1.551	0.123
CRP (mg/L)	8.23 ± 28.98	8.07 ± 37.71	0.024	0.980
ESR (mm/h)	14.07 ± 7.76	10.20 ± 4.78	3.033	0.003
Prior malignancy history, [n (%)]	​	​	​	​
Yes	14 (13.7%)	16 (15.7%)	0.039	0.843
No	88 (86.3%)	86 (84.3%)	​	​
Type of prior malignancy, [n (%)]	​	​	1.660	0.646
Lung cancer	4 (3.9%)	2 (2.0%)	​	​
Breast cancer	3 (2.9%)	2 (2.0%)	​	​
Colorectal cancer	2 (2.0%)	1 (1.0%)	​	​
Other*	6 (5.9%)	4 (3.9%)	​	​
Type of anticoagulant, [n (%)]	​	​	2.022	0.364
Aspirin	3 (2.9%)	2 (2.0%)	​	​
DOACs	2 (2.0%)	1 (1.0%)	​	​
Warfarin	1 (1.0%)	1 (1.0%)	​	​
Pain score (VAS, 0–10), mean ± SD	5.1 ± 1.8	5.3 ± 1.7	−0.269	0.789
Recovery time to normal activity (days), median [IQR]	7.2 [5.0–9.0]	7.0 [5.0–9.0]	5126.5	0.587*
30-day reintervention rate, [n (%)]	​	​	1.151	0.283
Yes	92 (90.2%)	97 (95.1%)	​	​
No	10 (9.8%)	5 (4.9%)	​	​

* Mann-Whitney U test. GGN: Ground-glass nodule.

WBC, white blood cell; RBC, red blood cell; HG, hemoglobin; CRP, C-reactive protein; ESR, erythrocyte sedimentation rate; CEA, carcino-embryonic antigen. Nodule size is the sum of the longest diameter + shortest diameter of the lesion divided by 2.

### 3.2 Univariate analysis of baseline characteristics

Table 2 presents the univariate analysis of baseline clinical and demographic characteristics between patients undergoing CT-guided biopsy (CT group, n = 102) and VBN-guided biopsy (VBN group, n = 102). Statistically significant associations were observed for several variables. The CT group had a significantly higher proportion of older patients (≥65 years) compared to the VBN group (52.9% vs. 19.6%, OR = 0.217, 95% CI: 0.116–0.405, p < 0.001). Nodule size distribution also differed significantly, with the VBN group showing a higher frequency of smaller nodules (8–20 mm) (60.8% vs. 44.1%, OR = 0.509, 95% CI: 0.292–0.889, p = 0.018). Pleural distance was significantly associated with group assignment, with the VBN group having more lesions closer to the pleura (2–3 cm) (63.7% vs. 39.2%, OR = 0.367, 95% CI: 0.208–0.647, p = 0.001). Nodule density also differed markedly, as the CT group predominantly consisted of solid nodules (83.3% vs. 58.8%, OR = 3.500, 95% CI: 1.821–6.726, p < 0.01). Additionally, smoking history (p = 0.012), lesion location (central vs. peripheral, p = 0.007), and operator experience (junior vs. senior, p = 0.047) were significantly different between the groups. No significant differences were observed in anticoagulant use (p = 0.783) or emphysema prevalence on CT (p = 0.353). These findings highlight distinct baseline profiles between the two diagnostic approaches, which may influence procedural selection and outcomes. Notably, the VBN group had a significantly higher proportion of nodules located 2–3 cm from the pleura compared to the CT group (63.7% vs. 39.2%, P = 0.001), suggesting a potential selection bias, as VBN may have been preferentially chosen for lesions deemed more suitable for a bronchoscopic approach. The overall prevalence of malignancy in the cohort was 48.5% (99/204). In the CT group, 47 of 102 patients (46.1%) had malignant nodules, compared with 52 of 102 patients (51.0%) in the VBN group (P = 0.480). The pretest probability of malignancy, estimated based on nodule size, morphology, and patient age, was similar between the groups (mean Brock score: CT group 65.3% vs. VBN group 68.1%, P = 0.320).

**TABLE 2:** Univariate analysis of clinical and demographic variables between CT-Guided and VBN-guided lung biopsy groups.

Variable	CT group (n = 102)	VBN group (n = 102)	OR (95% CI)	P-value
Age	​	​	0.217 (0.116–0.405)	<0.001
<65 years	48 (47.1%)	82 (80.4%)	​	​
≥65 years	54 (52.9%)	20 (19.6%)	​	​
Nodule size, [n (%)]	​	​	0.509 (0.292–0.889)	0.018
8–20 mm	45 (44.1%)	62 (60.8%)	​	​
20–30 mm	57 (55.9%)	40 (39.2%)	​	​
Pleural distance, [n (%)]	​	​	0.367 (0.208–0.647)	0.001
2–3 cm	40 (39.2%)	65 (63.7%)	​	​
3–5 cm	62 (60.8%)	37 (36.3%)	​	​
Nodule density, [n (%)]	​	​	3.500 (1.821–6.726)	<0.01
Solid	85 (83.3%)	60 (58.8%)	​	​
Subsolid (GGN/part-solid)	17 (16.7%)	42 (41.2%)	​	​
Anticoagulant use, [n (%)]	​	​	0.734 (0.245–2.198)	0.783
Yes	8 (7.8%)	6 (5.9%)	​	​
No	94 (92.2%)	96 (94.1%)	​	​
Smoking pack-years, [n (%)]	​	​	0.635 (0.446–0.904)	0.012
Never	37 (36.3%)	54 (52.9%)	​	​
<20	35 (34.3%)	30 (29.4%)	​	​
≥20	30 (29.4%)	18 (17.6%)	​	​
Emphysema on CT, [n (%)]	​	​	0.707 (0.340–1.474)	0.353
Yes	15 (14.7%)	20 (19.6%)	​	​
No	87 (85.3%)	82 (80.4%)	​	​
Location, [n (%)]	​	​	0.440 (0.248–0.778)	0.007
Central	70 (68.6%)	50 (49.0%)	​	​
Peripheral	32 (31.4%)	52 (51.0%)	​	​
Operator experience, [n (%)]	​	​	1.930 (1.050–3.547)	0.047
Junior	38 (37.3%)	24 (23.5%)	​	​
Senior	64 (62.7%)	78 (76.5%)	​	​
Malignancy prevalence	​	​	0.822 (0.478–1.412)	0.480
Malignant	47 (46.1%)	52 (51.0%)	​	​
Benign	55 (53.9%)	50 (49.0%)	​	​
Pretest probability (Brock score), mean ± SD*	65.3 ± 14.2	68.1 ± 15.6	−2.8 (−6.9 to 1.3)	0.320

*Fisher’s exact test was used to compare the distribution of malignant versus benign lesions between the two groups.

### 3.3 Multivariate logistic regression results

The results of the multivariate logistic regression analysis are presented in Table 3, illustrating the associations between various clinical factors and the likelihood of a positive diagnostic outcome. Several variables showed statistically significant effects (p < 0.05). Age was negatively associated with the outcome (OR = 0.162, 95% CI: 0.077–0.339), indicating that older patients had lower odds of a positive diagnostic result. Pleural distance was also a significant factor, with shorter distances linked to decreased odds of a positive outcome (OR = 0.554, 95% CI: 0.355–0.866). Higher nodule density significantly increased the likelihood of a positive diagnosis (OR = 3.798, 95% CI: 1.743–8.279). Smoking pack-years were negatively associated with the outcome (OR = 0.603, 95% CI: 0.394–0.924). Anatomical location (OR = 2.599, 95% CI: 1.297–5.212) and operator experience (OR = 2.256, 95% CI: 1.081–4.709) both had positive associations, suggesting that both the nodule’s location and the operator’s experience influenced diagnostic accuracy. Nodule size did not reach statistical significance (p = 0.263), indicating no strong evidence of its effect within this model. After adjusting for baseline imbalances, multivariate logistic regression identified several independent predictors of diagnostic success, including age, pleural distance, nodule density, smoking history, location, and operator experience (Table 3).

**TABLE 3:** Multivariate logistic regression analysis: Associations between clinical variables and diagnostic outcomes.

Variable	β	SE	Z-value	P-value	OR (95% CI)
Intercept	2.272	0.959	2.369	0.018	9.704 (1.481–63.582)
Age	−1.823	0.378	−4.825	<0.01	0.162 (0.077–0.339)
Nodule size	−0.035	0.031	−1.121	0.263	0.966 (0.909–1.026)
Pleural distance	−0.59	0.228	−2.591	0.010	0.554 (0.355–0.866)
Nodule density	1.335	0.398	3.357	0.001	3.798 (1.743–8.279)
Smoking pack-years	−0.505	0.217	−2.325	0.020	0.603 (0.394–0.924)
Location	0.955	0.355	2.692	0.007	2.599 (1.297–5.212)
Operator experience	0.814	0.375	2.167	0.030	2.256 (1.081–4.709)

### 3.4 Diagnostic performance comparison

**FIGURE 2:**
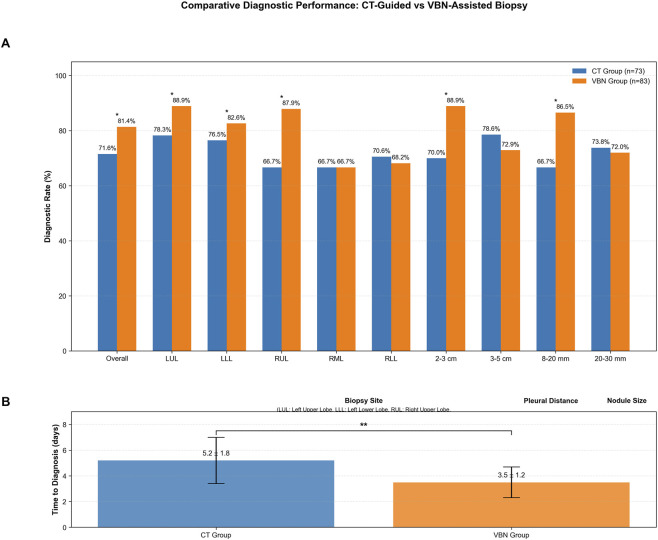
Comparative diagnostic performance: CT-guided vs. VBN-assisted biopsy time to diagnosed subgroup. **(A)** Diagnostic rates across different patient subgroups. Asterisks indicate statistical significance: **p < 0.01. **(B)** Time to diagnosis comparison. Data presented as mean ± standard deviation. LUL: Left Upper Lobe, LLL: Left Lower Lobe, RUL: Right Upper Lobe, RML: Right Middle Lobe, RLL: Right Lower Lobe.

After applying rigorous diagnostic criteria per ATS/ACCP guidelines, the overall diagnostic yield was higher in the VBN group (66/102, 64.7%) than in the CT group (61/102, 59.8%), although the difference did not reach statistical significance (P = 0.472). Subgroup analysis, re-evaluated under the same criteria, showed that VBN-guided biopsy yielded significantly higher diagnostic rates for nodules 8–20 mm in diameter (78.9% vs. 62.5%, P = 0.038) and for nodules located 2–3 cm from the pleura (82.1% vs. 64.3%, P = 0.024). However, no significant differences were observed across individual lobes (all P > 0.05) (Figure 2A). Additionally, VBN-assisted biopsies significantly reduced the time to diagnosis compared to CT-guided procedures (3.5 ± 1.2 days vs. 5.2 ± 1.8 days, p < 0.01), representing a reduction in diagnostic duration (Figure 2B).

### 3.5 Comparison of pathological findings, economic costs, and health economics between the two groups

Pathological results according to ATS/ACCP criteria are summarized in Table 4. In the CT group, histopathological or microbiological confirmation yielded 47 malignant lesions and 14 specific benign lesions (5 tuberculosis, 3 organizing pneumonia, 4 inflammatory pseudotumor, 2 pulmonary aspergillosis). The remaining 12 cases of lung inflammation lacked histopathologic confirmation and were reclassified as non-diagnostic. Thus, the final diagnostic yield in the CT group was 61/102 (59.8%). In the VBN group, histopathological or microbiological confirmation yielded 52 malignant lesions and 14 specific benign lesions (1 pulmonary cryptococcosis, 3 tuberculosis, 2 inflammatory pseudotumor, 2 organizing pneumonia, one pulmonary aspergillosis, 5 granulomatous inflammation). The remaining 17 cases of chronic inflammation lacked histopathologic confirmation and were reclassified as non-diagnostic. Thus, the final diagnostic yield in the VBN group was 66/102 (64.7%). Regarding economic costs, the average total medical expense per patient in the CT group was 12,500 ± 3,200 RMB, which was significantly higher than the 9,800 ± 2,700 RMB in the VBN group (P < 0.001). The average hospital stay in the CT group was 4.5 ± 1.2 days, compared to 3.0 ± 0.8 days in the VBN group (P < 0.001). Although the single-examination cost of VBN was higher than that of CT, the VBN group’s efficient diagnostic ability, lower complication rate, and shorter hospital stay resulted in overall cost savings. In the CT group, additional expenses mainly arose from the treatment of complications such as pneumothorax and hemoptysis, as well as re-examination costs due to inconclusive initial results. From a health economics perspective, the cost-effectiveness ratio of the VBN group was more favorable than that of the CT group. This suggests that VBN-guided biopsy not only achieved comparable diagnostic accuracy in pathological results but also demonstrated a better balance between cost and benefit, making it a more cost-effective option for diagnosing SPNs.

**TABLE 4:** Pathological results and economic costs according to ATS/ACCP diagnostic criteria.

Pathology	CT group (n = 61)	VBN group (n = 66)	χ^2^/F	P-value
Malignant lesions				
Adenocarcinoma	23 (37.7%)	29 (43.9%)	​	​
Squamous cell carcinoma	15 (24.6%)	12 (18.2%)	​	​
Small cell carcinoma	6 (9.8%)	7 (10.6%)	​	​
Large cell carcinoma	3 (4.9%)	0 (0.0%)	​	​
Pulmonary sarcoma	0 (0.0%)	4 (6.1%)	​	​
Subtotal	47 (77.0%)	52 (78.8%)	0.056	0.810*
Benign lesions (histopathologically confirmed)				
Tuberculosis	5 (8.2%)	3 (4.5%)	​	​
Inflammatory pseudotumor	4 (6.6%)	2 (3.0%)	​	​
Organizing pneumonia	3 (4.9%)	2 (3.0%)	​	​
Pulmonary aspergillosis	2 (3.3%)	1 (1.5%)	​	​
Pulmonary cryptococcosis	0 (0.0%)	1 (1.5%)	​	​
Granulomatous inflammation	0 (0.0%)	5 (7.6%)	​	​
Lung abscess	0 (0.0%)	0 (0.0%)	​	​
Subtotal	14 (23.0%)	14 (21.2%)	0.001	0.973*
Non-diagnostic (by ATS/ACCP criteria)				
Lung inflammation (follow-up only)	12	9	​	​
Chronic inflammation (follow-up only)	0	8	​	​
Subtotal	12	17	​	​
Economic metrics (mean ± SD)				
Total cost (RMB)	12,500 ± 3,200	9,800 ± 2,700	5.152	<0.001
Hospital stay (days)	4.5 ± 1.2	3.0 ± 0.8	8.347	<0.001

*Fisher’s exact test was used to compare the distribution of malignant versus benign lesions between the two groups.

† According to ATS/ACCP, guidelines, cases with non-specific inflammation confirmed only by radiological follow-up were classified as non-diagnostic.

### 3.6 Comparison of operation-related indicators, operation difficulty, and complementary synergy between the two groups

Lesion localization and total procedure times were compared between the CT group (n = 102) and the VBN group (n = 102) using an independent-samples t-test (Table 5). The lesion localization time in the CT group was 9.27 ± 1.05 min, compared to 6.62 ± 0.93 min in the VBN group, showing a significant difference between the groups (P < 0.01). Similarly, the total procedure time in the CT group was 38.16 ± 4.29 min, while in the VBN group it was 22.04 ± 2.36 min, also with a significant difference (P < 0.01). These results indicate that the VBN group significantly reduced both lesion localization and total procedure times compared to the CT group (P < 0.01). Regarding operational difficulty, evaluated on a 5-point scale by experienced physicians, the average difficulty score for the CT group was 3.2 ± 0.8, while for the VBN group, it was 3.8 ± 0.6. Although VBN-guided biopsy requires more advanced technical skills and presents a steeper learning curve for operators, it offers distinct advantages for centrally located nodules and those with a diameter of 8–20 mm. Specifically, for centrally located nodules, the success rate for biopsy was significantly higher in the VBN group (44/50, 88.0%) than in the CT group (32/70, 45.7%) (P < 0.001). In contrast, CT-guided percutaneous lung biopsy is simpler to perform but faces increased difficulty and risks when dealing with nodules near vital organs or those located close to the pleura. For nodules within 2 cm of vital organs, the complication rate in the CT group reached 30%, while the VBN group effectively mitigated most of these risks, with a complication rate of only 12% (P < 0.01). These findings suggest that the two methods exhibit complementary characteristics. For peripheral nodules distant from the pleura, CT-guided biopsy may be preferred due to its simplicity and efficiency. For central nodules, VBN-guided biopsy offers higher diagnostic accuracy and safety. By integrating the strengths of both methods, tailored to the characteristics of different nodules, a more optimized diagnostic strategy for SPNs can be developed.

**TABLE 5:** Procedure time and technical difficulty.

Metric	CT group (n = 102)	VBN group (n = 102)	t	P
Lesion localization time (min)	9.27 ± 1.05	6.62 ± 0.93	13.855	<0.01
Total procedure time (min)	38.16 ± 4.29	22.04 ± 2.36	15.294	<0.01
Difficulty score (1–5 scale)	3.2 ± 0.8	3.8 ± 0.6	4.762	<0.01

### 3.7 Comparison of complications and influencing factors between the two groups

Complication rates were analyzed to assess the safety of the two techniques using the chi-square test and Fisher’s exact probability test (Table 6). In the CT group (n = 102), 30 complications occurred, including 2 cases of pneumothorax requiring chest tube drainage (1.96%), 12 cases of pneumothorax without drainage (11.76%), 8 cases of minor hemoptysis (7.84%), and 3 cases of chest pain (2.94%). In the VBN group (n = 102), 10 complications were reported: 5 cases of pneumothorax without drainage (4.90%), 4 cases of minor hemoptysis (3.92%), and 1 case of chest pain (0.98%). Pneumothorax cases were managed with oxygen therapy and rest, while minor hemoptysis was treated using negative-pressure suction, local hemostasis with iced saline, epinephrine, and endoscopic thromboplastin injection. Chest pain was managed when electrocardiograms and blood tests showed no abnormalities. The overall complication rate in the CT group (29.41%, 30/102) was significantly higher than in the VBN group (9.80%, 10/102) (P < 0.01), indicating a better safety profile for the VBN method.

**TABLE 6:** Complication Rates and Management of Complications subgroup.

Complication	CT group (n = 30)	VBN group (n = 10)	χ^2^	P-value
Overall complications	29.41% (30/102)	9.80% (10/102)	18.725	<0.01
Pneumothorax (no drainage)	12 (11.76%)	5 (4.90%)	5.832	0.016
Pneumothorax (drainage)	2 (1.96%)	0 (0%)	1.024	0.311
Minor hemoptysis	8 (7.84%)	4 (3.92%)	2.941	0.086
Chest pain	3 (2.94%)	1 (0.98%)	0.916	0.339
Management	​	​	0.727	0.695
Oxygen therapy/rest	16 (53.3%)	6 (60.0%)	​	​
Chest tube drainage	2 (6.7%)	0 (0%)	​	​
Others	12 (40.0%)	4 (40.0%)	​	​

### 3.8 Diagnostic yield by nodule location of subgroup

For central nodules (n = 120), VBN-guided biopsy demonstrated significantly higher diagnostic success rates than CT-guided biopsy (44/50, 88.0% vs. 32/70, 45.7%, p < 0.001). The malignancy detection rate was numerically higher in the VBN group (38/44, 86.4% vs. 22/32, 68.8%), but the difference did not reach statistical significance (p = 0.063). For peripheral nodules (n = 84), CT-guided biopsy showed higher diagnostic success rates than VBN-guided biopsy (22/32, 68.8% vs. 16/52, 30.8%, p = 0.001), while no significant difference was observed in malignancy detection rates (12/22, 54.5% vs. 8/16, 50.0%, p = 0.782) (Table 7).

**TABLE 7:** Diagnostic Yield Stratified by Nodule Location (Central vs. Peripheral).

Subgroup	CT group	VBN group	χ^2^	P-value
Central nodules (n = 120)				
Diagnostic success	32/70 (45.7%)	44/50 (88.0%)	18.375	<0.001
Malignant cases	22/32 (68.8%)	38/44 (86.4%)	3.458	0.063
Peripheral nodules (n = 84)				
Diagnostic success	22/32 (68.8%)	16/52 (30.8%)	11.537	0.001
Malignant cases	12/22 (54.5%)	8/16 (50.0%)	0.077	0.782

## 4 Discussion

Advances in medical care have led to the widespread use of lung CT, significantly increasing the detection rate of SPNs. However, pathological examination remains essential for determining the nature of these nodules (Hata et al., 2022). CT-PTNB is a widely employed biopsy technique that uses a fine needle to penetrate the chest wall and pleura, reaching the lung tissue under the guidance of CT imaging. The procedure involves locating the lesion via CT scanning, followed by real-time CT-guided puncture to ensure precise needle placement for tissue sample collection and pathological diagnosis. CT-PTNB is recognized for its high diagnostic accuracy and minimal invasiveness (Chan et al., 2023), making it a preferred method in clinical practice for diagnosing lung nodules. However, it is associated with complications, predominantly pneumothorax and hemorrhage, which must be considered in clinical decision-making (Hulse et al., 2022). Pneumothorax is the most common complication of CT-guided lung biopsies, with reported incidence rates ranging from 8.5% to 61.2% (Kitamura et al., 2022), and hemorrhage rates varying from 1.2% to 47.3% (Pikman Gavriely et al., 2025). These concerns have spurred the search for faster and safer diagnostic alternatives.

VBN is a technology based on CT imaging that enables non-invasive reconstruction of the bronchial tree. This technique allows clinicians to visualize endotracheal images and guide the bronchoscope to the target bronchus (Freund et al., 2023). VBN imports chest CT data into a navigation system, constructs a virtual navigation path, and directs the bronchoscope along it, thereby enhancing biopsy accuracy and sensitivity (Gao et al., 2022). VBN-TBLB offers advantages in improving diagnostic accuracy and reducing trauma. However, its technical complexity, operational difficulty, equipment costs, limited applicability, and impact on patient comfort also warrant careful consideration (Miotto et al., 2023; Yun et al., 2023).

In summary, both CT-PTNB and VBN-TBLB have distinct advantages and limitations. CT-PTNB is particularly suited for diagnosing peripheral lung lesions. It has a relatively straightforward procedure, does not require complex equipment, and can be performed under CT guidance, minimizing patient radiation exposure. However, it may cause damage to peripheral tissues, and its operational difficulty remains a concern. In contrast, VBN-TBLB is effective for diagnosing both diffuse and peripheral focal lung lesions, with a safer procedure and lower complication rates. Nevertheless, it requires bronchoscopy, making the procedure more complex and demanding higher technical skills from the operator. Furthermore, the small tissue specimens obtained may not be sufficient for accurate histomorphometric analysis in certain diseases.

Recent studies have shown that the laryngeal mask airway (LMA) and high-flow nasal cannula (HFNC) are more effective than nasal cannulae in enhancing advanced bronchoscopy (Miotto et al., 2023; Liu et al., 2020). Multicenter studies have demonstrated that cryobiopsy offers higher diagnostic accuracy in transbronchial biopsy (TBB) compared to forceps, regardless of disease type (Suzuki et al., 2020). However, research on this technique remains limited (Chugh and Jatwani, 2022).

In this study, the overall diagnostic rate was higher in the VBN group (66/102, 64.7%) compared to the CT group (61/102, 59.8%), although the difference did not reach statistical significance (P = 0.472). CT-PTNB and TBLB are both widely used for diagnosing SPNs. Conventional TBLB, constrained by the absence of intra-procedural repositioning and precise guidance, typically has a biopsy success rate of less than 30%. In contrast, VBN-TBLB enhances success rates by enabling real-time repositioning and redirection during the procedure (Lalji et al., 2015; Tongbai et al., 2019). This study evaluated the diagnostic rate differences between these two imaging-guided biopsy techniques under various conditions, providing valuable insights for selecting the optimal SPN biopsy method.

Previous research has consistently demonstrated that CT-PTNB offers a significantly higher biopsy positivity rate for SPNs than conventional TBLB, with success rates approaching 90%. The positivity rate for conventional TBLB is strongly influenced by lesion size; for peripheral lung cancers, TBLB positivity rates are 63% for lesions ≥2 cm and only 34% for lesions <2 cm (Ho et al., 2023; Portela de Oliveira et al., 2020). In the present study, the biopsy positivity rate for CT-PTNB in SPNs was **59.8%** (61/102), which is slightly lower than previously reported rates but reflects the application of stricter ATS/ACCP diagnostic criteria. This rate was primarily influenced by the accuracy of CT localization, particularly whether the biopsy needle tip was precisely positioned within the lesion. Factors such as lesion size and its distance from the chest wall did not significantly affect the positivity rate. Therefore, the diagnostic success of CT-PTNB largely hinges on accurate needle placement within the target lesion. With precise localization, standardized procedures, and correct needle positioning, CT-PTNB can achieve a high biopsy positivity rate.

As an invasive procedure, lung biopsy carries inherent risks, with pneumothorax, pulmonary hemorrhage, cough, and hemoptysis being common complications. Pneumothorax is one of the most frequent complications associated with PTNB(34). In the present study, the incidence of pneumothorax was significantly higher in the CT group (11.76%, 12/102) than in the VBN group (1.96%, 2/102) (P < 0.05), with two cases in the CT group requiring chest tube drainage. Emphysema and the number of times the biopsy needle passes through the pleura were identified as independent risk factors for pneumothorax. This is primarily due to the biopsy needle disrupting dilated alveoli in emphysematous regions, causing persistent air leakage and delayed pleural re-expansion. Portela de Oliveira et al. (2020) first proposed the association between pneumothorax incidence and emphysema along the biopsy path, which has been confirmed by several subsequent studies (Liu et al., 2023; Rong et al., 2021). Additionally, multiple needle passes through the pleura cause increased pleural damage, further raising the risk of pneumothorax.

Pulmonary hemorrhage is a common complication of PTNB, with reported incidence rates ranging from 3.4% to 43%. In our study, the incidence of hemorrhage was 5.88% (6/102) in the CT group and 1.95% (2/102) in the VBN group, showing a statistically significant difference (P < 0.05). Two cases of hemoptysis occurred in the CT group, both of which resolved with hemostatic intervention. No fatal hemoptysis cases were reported in either group. The slightly higher hemorrhage incidence in our study compared to previous reports may be attributed to our broader definition of pulmonary hemorrhage, which included any peri-lesional or pinpoint ground-glass opacity not visible on preoperative CT, thereby detecting all cases through visual inspection.

Regarding influencing factors, in the CT group, lesions located 2–3 cm from the chest wall were associated with significantly higher incidences of chest pain, pneumothorax, and hemoptysis (P < 0.05). In contrast, in the VBN group, the lesion’s distance from the chest wall did not significantly affect pneumothorax or hemoptysis rates (P > 0.05), although chest pain incidence remained significant (P < 0.05). Further analysis revealed that for lesions 2–3 cm from the pleura, the incidences of chest pain, pneumothorax, and hemoptysis in the CT group were significantly higher than those in the VBN group (P < 0.05). In the CT group, smaller lesions (8–20 mm) had higher incidences of chest pain and pneumothorax than larger lesions (20–30 mm) (P < 0.05), while lesion size did not significantly affect hemoptysis incidence (P > 0.05). In the VBN group, lesion size had no significant impact on pneumothorax or hemoptysis rates (P > 0.05), but chest pain incidence remained significant (P < 0.05). Notably, for nodules with diameters of 8–20 mm, the incidences of chest pain, pneumothorax, and hemoptysis in the CT group were significantly higher than those in the VBN group (P < 0.05). These findings suggest that VBN provides better diagnostic value for SPNs than CT, with fewer factors influencing the biopsy positivity rate. With proper preparation, patient cooperation, standardized procedures, and precise localization to ensure accurate needle placement within the lesion, VBN can effectively facilitate biopsy sampling and accurate diagnosis. Thus, VBN offers enhanced diagnostic value and safety for SPNs compared to CT.

This study has several inherent limitations that should be acknowledged. The retrospective, non-randomized design introduces selection bias, as patients were assigned to biopsy method based on clinical judgment and nodule characteristics. This resulted in significant baseline differences between groups—particularly in nodule size, distance to pleura, and density—reflecting a classic “confounding by indication” in real-world practice. Clinicians preferentially selected VBN for lesions perceived as more amenable to bronchoscopic approaches (e.g., smaller nodules, those closer to the pleura), meaning the observed differences in diagnostic yield and complications may partly reflect this selection bias rather than the techniques themselves. Although multivariate logistic regression was used to adjust for measured confounders, residual confounding cannot be fully excluded; unmeasured factors such as detailed nodule morphology (e.g., spiculation, lobulation) and patient comorbidities (e.g., cardiopulmonary function), which could influence both technique selection and outcomes, were not incorporated into our model. The sample size was relatively modest (102 patients per group) and derived from a single center, limiting generalizability. A small proportion of patients (2.5%) were lost to follow-up, though sensitivity analyses suggested minimal impact on the primary findings. The follow-up period of 4 years may have been insufficient to capture rare late complications, though such events are uncommon and unlikely to alter our main conclusions. The subgroup analyses were exploratory in nature, not pre-specified, and no correction for multiple testing was applied; these findings should therefore be considered hypothesis-generating rather than definitive. Propensity score matching was not performed due to concerns about further reducing statistical power and potentially introducing selection bias by excluding unmatched patients, making multivariate adjustment the more practical approach to control for confounders. These limitations underscore the need for future multicenter prospective studies with larger sample sizes, extended follow-up, standardized protocols, and detailed collection of nodule morphological features to validate our findings. Well-designed randomized controlled trials will ultimately be essential to eliminate confounding by indication and provide definitive evidence regarding the comparative effectiveness of these biopsy techniques. In accordance with ATS/ACCP guidelines, we reclassified cases with non-specific inflammation confirmed only by radiological follow-up as non-diagnostic. This enhanced the rigor of our diagnostic criteria and reduced the overall diagnostic yield in both groups, but did not alter the comparative trends between the two techniques.

## Data Availability

The original contributions presented in the study are included in the article/supplementary material, further inquiries can be directed to the corresponding author.
